# Priority effects drive strain-level community composition of honeybee gut microbiota

**DOI:** 10.1093/ismejo/wrag056

**Published:** 2026-03-17

**Authors:** Aiswarya Prasad, Gonçalo Santos-Matos, Alexandra Szigeti-Genoud, Florent Mazel, Philipp Engel

**Affiliations:** Department of Fundamental Microbiology, University of Lausanne, 1015 Lausanne, Switzerland; Department of Fundamental Microbiology, University of Lausanne, 1015 Lausanne, Switzerland; Department of Fundamental Microbiology, University of Lausanne, 1015 Lausanne, Switzerland; Department of Fundamental Microbiology, University of Lausanne, 1015 Lausanne, Switzerland; Department of Fundamental Microbiology, University of Lausanne, 1015 Lausanne, Switzerland

**Keywords:** symbiosis, insect gut, competition, colonization order, community composition, interspecific interactions, 16S rRNA PacBio sequencing, Bifidobacteria, Lactobacillus

## Abstract

Gut microbial communities often differ at the strain level among individual hosts, but the mechanisms driving this variation remain poorly understood. One potential factor is “priority effects”, a process in which differences in the timing and order of microbial colonization influence subsequent community assembly (“first come, first served” dynamics). We hypothesize that priority effects operate at the strain level within species, where closely related bacteria exhibit niche overlap, and that these dynamics can lead to community divergence even under similar environmental conditions. We tested these predictions, using the gut microbiota of honeybees, which harbor conserved microbial communities that differ in strain composition among individual bees. We sequentially colonized microbiota-depleted honeybees with two distinct microbial communities composed of the same 12 core microbiota species but different strains, ensuring that individuals shared species-level composition but differed at the strain level. We found that firstcomer strains consistently dominated the resulting communities, suggesting strong priority effects. Dropout experiments in which the firstcomer strain of a species was removed led to only partial increases in the colonization success of the conspecific latecomer, suggesting that both intra- and inter-species interactions contribute to priority effects. Our findings highlight the significant role of priority effects in strain-level community assembly and reveal their influence in shaping the specialized gut microbiota of honeybees, with important implications for the development of probiotic strategies in beekeeping.

## Introduction

Gut microbial communities exhibit substantial bacterial diversity, particularly at the strain level. Closely related strains can stably coexist within the same host [1–4]. But different hosts typically harbor distinct strain profiles, even when closely related or exposed to the same environment [2, 4]. These differences in strain-level composition can substantially influence the functional potential of the microbiota and how they interact with and benefit the host [5]. Studying the mechanisms that underlie strain-level differences across individuals are hence fundamental to our understanding of the relationship between hosts and their gut microbiota.

An important mechanism of microbial community assembly is environmental filtering. This occurs when differences in physicochemical conditions, such as nutrient availability, pH, transit time, oxygen levels, and immune system activity determine which microbes can colonize a given environment based on their functional traits. These deterministic factors are well studied in the gut microbiota [6–8], but other mechanisms are less understood. These include dispersal limitation, or the ability of microbes to reach and colonize specific hosts (due to spatial barriers such as geography) [9, 10], and stochastic processes, random colonization events that occur independently of microbial traits or environmental conditions [11, 12]. One such stochastic phenomenon is priority effects [13], where the timing and order of arrival shape the resulting community composition [11, 14]. Microbes that arrive earlier can occupy and/or alter the environment, affecting the colonization success of subsequent arrivals. Although microbiome studies have investigated priority effects between bacterial species [15–19], few studies have documented priority effects at the strain level and in the context of a multi-species community [20–22]. This is surprising given that contemporary coexistence theory [23–27] predicts priority effects should be stronger between competitors with small fitness differences and overlapping niches. The presence of a complex, multi-species community can further amplify these effects, as interactions with other community members may impose additional constraints on the niches of each strain in the community. Hence, we hypothesize that priority effects should be an important factor in shaping gut microbiome composition at the strain level leading to differences across individuals.

Western honeybees (*Apis mellifera*) are an excellent model for studying the processes underlying community assembly, particularly priority effects. Honeybees harbor a relatively simple and stable gut microbiota, composed of a few host-specific bacteria that include multiple closely related species and strains [1, 28, 29]. Whereas most species are consistently present and often co-occur in individual bees, strain-level variation is more individualized; in other words, conspecific strains (i.e. strains of the same species) tend to segregate between individual bees, even within the same colony [1]. Priority effects early in life, when newly emerged adult bees acquire microbiota from their nestmates, have been proposed as one possible mechanism for the observed patterns [1] and a recent study has shown that the order of arrival matters for strains of the bee gut symbiont *Snodgrassella alvi* [22].

Here, we experimentally tested the role of priority effects in the assembly of more complex communities at the strain level by inoculating microbiota-deprived (MD) bees under laboratory conditions with two microbial communities administered 3 days apart. Each community consisted of the same 12 species, but different strains. We also conducted dropout experiments, in which a single strain from a subset of the 12 species was excluded from the firstcomer community to test whether this increased the colonization success of its conspecific strain in the latecomer community. Our results show that priority effects are an important ecological process shaping gut microbiota diversity at the strain level and that they are influenced by both within- and between species interactions.

## Materials and methods

### Experimental design and pilot experiment

**Figure 1:**
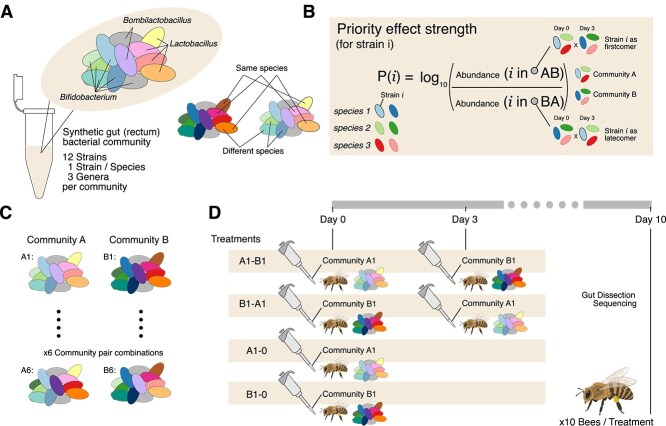
Experimental design and community selection*.* (A) Schematic of the multi-species bacterial communities used for the experiments. Each community consisted of 12 species, two of *Bombilactobacillus*, four of *Lactobacillus*, and six of *Bifidobacterium*. (B) Illustration of the approach used to measure the priority effect for a given strain *i*. (C) Schematic representing community pairs A and B made in six different combinations (1–6) by shuffling strains between them. (D) Illustration of the experimental design to test each community combination (here, community 1). Bee illustrations in this figure were created with BioRender.com.

To test the role of priority effects in shaping strain-level community assembly in the honeybee gut microbiota, we designed an experiment where strains from the same species were alternatively introduced as “firstcomers” or “latecomers” some time apart. If priority effects were strong, we expected the firstcomer to predominate in the community. We assembled multi-species synthetic communities comprised of the same 12 species but harboring a different strain for 9 of the 12 species. All species belonged to either *Bifidobacterium*, *Lactobacillus,* or *Bombilactobacillus,* which are the predominant genera in the distal hindgut (rectum) of the Western honeybee (*A. mellifera*) (Fig. 1A, Supplementary Table S1).

To inform the design and interpretation of the main experiment, we conducted a pilot study in which two communities composed of the same strains used in the main experiment were compared across 13 different treatments (Supplementary Fig. S1 and Supplementary Methods). The results of this pilot study indicated that a 3-day difference between firstcomer and latecomer strains led to patterns of colonization that differed from those observed when all strains were inoculated simultaneously (i.e. when no arrival-order advantage exists). This result suggests that priority effects are at play and that the observed patterns cannot be explained by competitive dominance of one of the strains. Moreover, this pilot study showed that it was not necessary to control for differences in bee age between firstcomer and latecomer inoculation or for length of colonization (see Supplementary Methods for details).

Informed by the results of the pilot experiment, in the main experiment, MD bees were inoculated on Day 0 with the firstcomer strains (e.g. “Community A1” in Fig. 1D) and subsequently challenged on Day 3 with the latecomer strains (e.g. “Community B1” in Fig. 1D). We chose these particular timepoints of inoculation based on our pilot experiment results and because they lie in the middle of the exponential growth phase of the bacteria during community assembly [30] and hence offer the opportunity to invade the community before it has reached carrying capacity.

Bees colonized with only one of the two communities at Day 0 served as positive controls to verify that the strains of a community successfully colonize in the absence of any other intervention. Reciprocal inoculation order ensured that the observed bias in community composition was not driven by one community being intrinsically more competitive than the other (Fig. 1D). Bacterial loads and community composition in the hindgut were measured on Day 10.

The strength of the priority effect for each strain within each community was quantified as the log ratio of its absolute abundance when inoculated into the community on Day 0 versus Day 3 (Fig. 1B). Abundance measurements from bees colonized with only a single community on Day 0 (positive controls) were excluded from this calculation. To replicate the measurement of priority effect at the strain level but under different community backgrounds, we made six community combinations by shuffling strains between community A and B such that in each combination, a given strain co-occurred with a different set of strains of the other 11 species (Supplementary Table S2, Fig. 1C). For species where more than one strain was available, strains were swapped in such a way that the background community of each strain was different by at least five strains. This can be visualized in Supplementary Table S2 by following the strains marked in red and blue.

For two *Bombilactobacillus* species (*B. mellifer*, *B. mellis*) and one *Bifidobacterium* species (*B. cornyforme*), the same strain was used in all communities, and for one Lactobacillus species (*L. melliventris*) we had by mistake included two strains in some of the communities (more detailed explanation in Supplementary Methods). So, we could only assess the priority effects for 8 of the 12 tested species in a reciprocal manner.

For each pair of community combinations, we included four treatments (A, B, AB and BA) (Fig. 1D). In treatments A and B, MD bees were only inoculated with one community at Day 0. These treatments served as controls as explained above. In AB and BA, the two communities were inoculated one after the other on Day 0 and Day 3, respectively (Fig. 1D). Each strain appeared in either treatment A or B of each community combination. Consequently, a strain present in community A, is a firstcomer in treatment AB and a latecomer in treatment BA. For each community combination and treatment, we analyzed 8–10 bees from the cage. This way, each strain was measured a total of about 60 times as a firstcomer and a latecomer each across the six different community combinations (Fig. 1C).

### Bacterial culturing and synthetic community assembly

A total of 22 strains belonging to the 12 selected species were used in this study. They were all isolated from the gut of adult bees of the Western honeybee (*A. mellifera*) either in this or in previous studies [31, 32]. We used pairwise average nucleotide identity to determine which strain belongs to which species (Supplementary Fig. S2). Strains for which no complete genome was available were re-sequenced using PacBio or Nanopore sequencing and all full-length 16S rRNA genes of each strain were extracted using barrnap [33] to ensure that all strains can be discriminated by at least one to two SNPs in at least one variant. Details about each strain can be found in Supplementary Table S1.

To assemble the six synthetic communities, the selected strains were grown on solid De Man–Rogosa–Sharpe agar (supplemented with 2% w/v fructose and 0.2% w/v L-cysteine-HCl) in petri dishes at 34°C under anaerobic conditions. After ~48 h of growth, each strain was harvested using a sterile loop and resuspended in 300 μL of a sterile solution of PBS + 20% glycerol. The solution was diluted to an OD_600_ value of 2 in PBS + 20% glycerol. To assemble the synthetic communities, 10 μL of each of the 12 strains were mixed into a sterile tube. This resulted in about 120 μL for each community combination (for dropout communities, sterile PBS + 20% glycerol solution was added instead of the dropped strain), to which 80 μL of PBS + 20% glycerol solution was added to make up a total of 200 μL of microbial solution for each community. This process ensured that each strain would be present in a quantity equivalent to a final OD_600_ value of 0.1. The final mixture for each community was thoroughly mixed and separated into 50 μL aliquots, which were flash frozen using liquid nitrogen and stored at −80°C until used.

### Gnotobiotic bee experiments

MD honeybees were obtained from *Apis mellifera carnica* colonies maintained at the University of Lausanne, as previously described [34]. More details about how MD bees were obtained and maintained can be found in the Supplementary Methods. One day after eclosion (here D0), bees were fed 5 μL of inoculum prepared from glycerol stock aliquots of the synthetic community, diluted in 450 μL of a 1:1 mixture of sterile sugar water (50% sucrose, w/v) and 1× PBS. All inoculations were performed at room temperature. After inoculation, bees were transferred to their respective sterilized 3D-printed cages (one per treatment), containing sterile pollen and sugar water tubes *ad libitum*. In each treatment, 10–12 bees were included per cage. For treatments involving a second inoculation on Day 3, feeding was repeated as done on Day 0. Finally, on Day 10, all surviving honeybees (8–10) were sacrificed and their hindguts were dissected, flash frozen using liquid nitrogen, and stored at −80°C until DNA extraction.

### DNA extraction and amplicon sequencing

DNA was extracted from dissected hindguts using a magnetic bead-based protocol optimized for high-throughput processing on the Opentrons OT-2 liquid-handling robot. Samples were homogenized in bead-beating tubes containing a mix of 1 mm glass beads and 0.1 mm zirconia beads, 750 μL 1× G2 buffer supplemented with lysozyme (100 mg/ml) in a Fast-Prep24 5G homogenizer (MP Biomedicals) at 6 m/s for 30 s twice. For lysis the homogenate was then incubated at 37°C for 30 min with shaking (900 rpm), followed by the addition of Proteinase K (20 mg/ml) and further incubation at 56°C for 1 h. For extracting DNA from the inocula fed to bees, 165 μL was used for bead-beating. Lysates were centrifuged, and 80 μL was transferred to a 96-well PCR plate. Extraction blanks were included as wells only containing nuclease-free water instead of gut homogenates. DNA purification was performed using CleanNGS magnetic beads (Clean NA #CNGS-0050) on an Opentrons OT-2 robot. Automated steps included magnetic bead binding, two 80% ethanol washes, drying, and elution in nuclease-free water. Eluates (30 μL) were collected on a new plate and stored at −20°C or −80°C. DNA concentrations were quantified using a Qubit fluorometer and the 1X dsDNA HS assay. 0.1x dilutions were made and stored on an additional plate and used for further steps.

### Quantification of total bacterial abundance

qPCR was performed on DNA extracts diluted to 0.1x to minimize the effect of contaminants. Primers targeting a 162 bp stretch of the V4 region of the 16S rRNA gene were used to quantify the entire bacterial community as described before [34], and as outlined in the Supplementary Methods.

### Library preparation and amplicon sequencing

For amplicon sequencing, multiplexed amplicon libraries were prepared using the “Amplification of Full-Length 16S Gene with Barcoded Primers for Multiplexed SMRTbell Library Preparation and Sequencing protocol” (Version 05). PCR amplification was performed using the KAPA HiFi HotStart ReadyMix (KAPA Biosystems) and a pre-prepared barcoded primer plate containing the forward and reverse primers 5′-GCATC/barcode/AGRGTTYGATYMTGGCTCAG-3′ and 5′-GCATC/barcode/RGYTACCTTGTTACGACTT-3′ in each well combined to result in 96 unique pairs as recommended in the protocol. Amplicons were diluted to 0.01x and quantified using qPCR as described in the following section and the Supplementary Methods. Using the results of the quantification, amplicons were pooled in equimolar quantities for library preparation using the SMRTbell Prep Kit 3.0. Library preparation and sequencing were carried out in the Next Generation Sequencing Platform of the University of Bern.

### Amplicon sequencing analysis to determine strain abundance

Raw reads from sequencing were processed using DADA2 v1.30.0 [35] to infer amplicon sequence variants (ASV) and their counts per sample as described before [36]. The detailed code used for this analysis is provided in the associated GitHub repository https://github.com/Aiswarya-prasad/2025_aprasad_PriorityEffects. The “removePrimers” function was used for primer removal and automatically re-orienting all reads in the forward direction. Reads were then filtered to only keep reads in the range of 1000–1600 bp. Finally, reads were dereplicated and denoised using a PacBio sequencing specific error model.

The inferred ASVs were matched with unique 16S rRNA gene variants of each strain and used to infer the counts per strain that would result in the observed ASV counts table generated by DADA2. This was done using the code (infer_species_counts.py) provided in the associated GitHub repository https://github.com/Aiswarya-prasad/2025_aprasad_PriorityEffects. To resolve strain-level composition from ASV counts, we implemented a matrix-based least-squares approach, leveraging known relationships between strains and their 16S rRNA gene copy profiles. We took this approach because each strain comprises several unique or non-unique ASVs and the counts of their ASVs are not equal even when they have the same copy number due to technical errors in amplification and sequencing (more details in Supplementary Methods).

Samples with low 16S rRNA gene copies (${C}_T$> 26.53 or undetermined) or sequencing depth (<100 known reads) were removed from further analyses (*n* = 15 of 365). The relative abundance of each strain was calculated as the ratio of the number of reads assigned to that strain and the total number of reads in that sample. The absolute abundance of each strain in a sample was estimated as the product of relative abundance and total copy number for that sample. The detection limit of each sample was the number of 16S rRNA gene amplicon copies yielding one read in that sample, specifically, the ratio of total gene copies and the number of reads sequenced. Any values of abundance below the detection limit were considered undetected and their value was set to 1 rather than 0 (for plotting on a log scale without errors).

## Statistical analyses

To compare community profiles across treatments, PERMANOVA was used. To do this, we used the Vegan (v2.6-4) package to obtain Bray–Curtis dissimilarities from the log-transformed sample vs. bacterial abundance matrix. The distance matrix was then used to make a PCoA and the adonis2 function was used to perform the PERMANOVA analysis. The effect size (ω^2^) was calculated using the adonis_OmegaSq function from the package https://github.com/Russel88/MicEco. The results are denoted as ω^2^ and *P* values.

The Wilcox rank sum test in R was used to compare differences between treatments as stated in the respective figure descriptions or references. The Benjamini**–**Hochberg method was used to correct for multiple comparisons where necessary. All tests were two-tailed and a *P* value <.05 was considered statistically significant.

For the generalized linear mixed model (GLMM), we used the “glmer” function from the lme4 package in R. The model was set up to predict the transformed absolute counts based on the arrival order, while accounting for random effects due to different community combinations. It was carried out using a Gamma distribution with a log link function, which is appropriate for modelling positive continuous data like counts.

## Results

### Priority effects influence community composition at the strain level

Most honeybees across treatments were successfully colonized by the inoculated community, (total bacterial abundances in Supplementary Fig. S3). The abundance of bacteria in the gut of honeybees at the sampling day (i.e. Day 10) was orders of magnitude higher than in the inoculum (fed on Day 0 and Day 3) and in non-inoculated MD bees fed sterile sugar water and pollen, indicating that the strains in the inoculum proliferated within the honeybee gut resulting in the assembled communities (Supplementary Fig. S4).

All 12 species were detected in most honeybee samples and the colonizing strains matched those present in the inocula of each treatment, confirming both the success of the experimental setup and the gnotobiotic status of our bees (Supplementary Figs S5 and S6). Total bacterial loads in each sample were not significantly correlated with the number of strains present (Spearman correlation ρ = 0.02, *P* = .219) (Supplementary Fig. S7), and the summed abundance for each species did not differ significantly whether one or two strains of the species were inoculated in the treatment (Supplementary Fig. S8). This indicates that the strains in each community reach carrying capacity and that latecomer strains compete in the niches occupied by the firstcomers rather than occupying additional empty niches.

**Figure 2:**
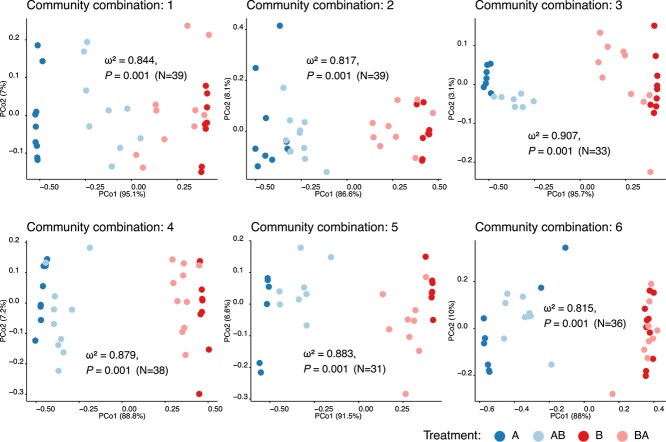
Community composition across samples. PCoA plots to visualize Bray–Curtis dissimilarity estimated from the matrix of log-transformed absolute abundance of bacterial strains across community combinations, with color indicating the treatment (A and B, community A and B on Day 0, respectively; AB, community A on Day 0 and community B on Day 3, and vice versa for BA).

To determine whether priority effects shape overall community composition we used the abundance of strains to estimate the Bray–Curtis dissimilarity between the samples of the four treatments in each community combination (~10 bees per treatment). PERMANOVA tests confirmed a strong (ω^2^ > 0.8) and significant (*P* < .05) effect of treatment across all community combinations. Communities AB (A inoculated at Day 0 and B at Day 3) closely resembled communities A, whereas communities BA resembled communities B across all replicates (Fig. 2). These differences were also visible across all replicates by metrics using either relative abundances or absence/presence of strains to compare the communities (Supplementary Fig. S9). These results indicate that the firstcomer strains dominated the communities and that the order of arrival of strains determines the overall community composition.

### Priority effects are consistently observed for all strains, but vary in strength

**Figure 3:**
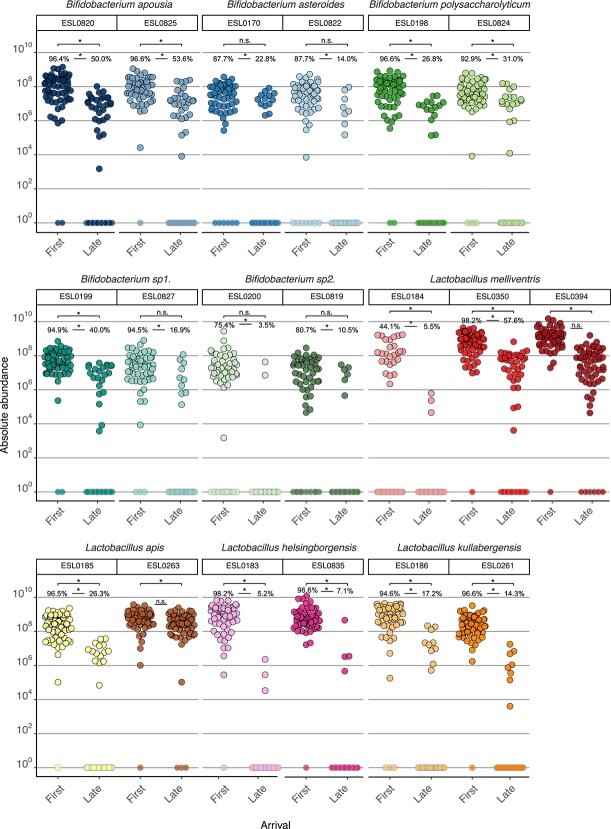
Abundance of each strain by arrival order. Absolute abundance of each strain (faceted by strain) under different arrival orders. “First” treatment: the focal strain was inoculated on Day 0 as part of a defined microbial community, and on Day 3 a second community (also containing the same conspecific strain) was added. “Late” treatment: the focal strain was instead introduced on Day 3 as part of the second community, into gnotobiotic bees that had already been inoculated on Day 0 with a community containing the same conspecific strain. Each plot includes all the treatments across the six community combinations. Result of Wilcoxon rank sum test (two-sided) of absolute abundance between groups in samples where the respective strain was detected is annotated on the topmost horizontal bar (*-*P* < .05), complete results of statistical test, including sample size per strain, are included separately (Supplementary Table S3). Points with gray outlines represent samples where the strain was not detected (below the detection threshold for that sample) and hence set to a value of 1. Numbers above each set of points indicate the percentage of samples in which the strain was detected and the line in between, the result of Fisher’s exact test on whether there was a significant difference in colonization success between groups (*-*P* < .05).

**Figure 4:**
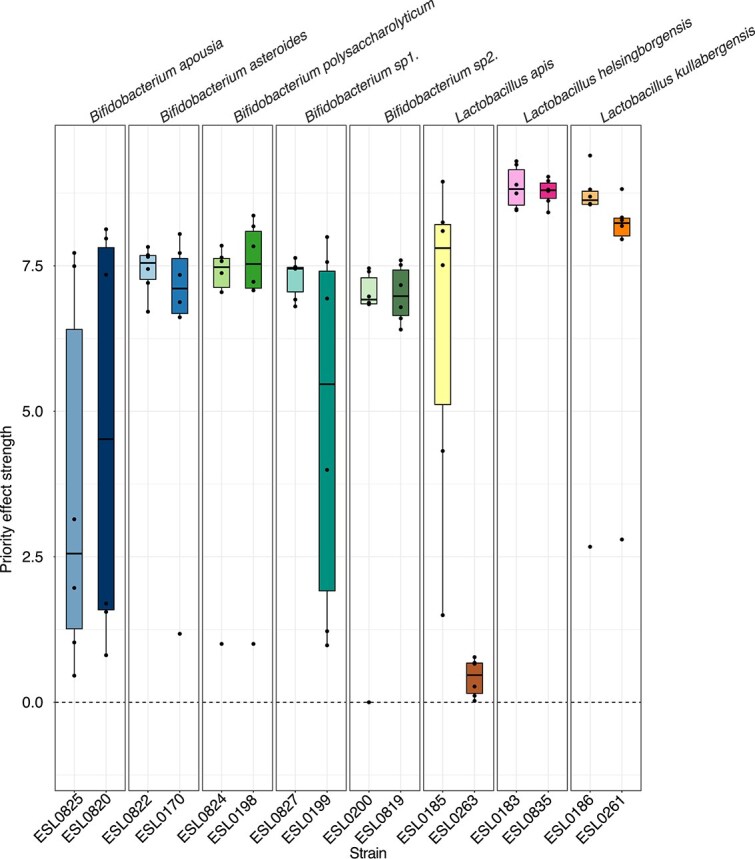
Strength of priority effect by strain across community combinations. Each point (black dot) represents a particular strain tested within one of the six community-pair combinations. The strength of the priority effect (expressed in log10 transformed values) is computed as shown in the formula in Fig. 1B using the median absolute abundance of a given strain in ~10 bees of each treatment AB and BA. Zero represents the value corresponding to no priority effect.

Strains of the same species may vary in the extent to which their niches overlap or in their ability to interfere with already established strains which can influence the strength of priority effects [13, 25, 37]. Most of the tested strains (17/19) showed a significantly lower frequency of colonization when introduced as latecomers (percentage of bees in which strain was detected, Fisher’s exact test *P* < .05, Fig. 3). Where sufficient data were available for meaningful comparisons, these strains also colonized to a lesser extent, exhibiting lower abundance when introduced as latecomers (Wilcoxon Rank sum test *P* < .05) as latecomers. However, we also observed variation in these patterns. Several strains were nearly undetectable when introduced as latecomers (e.g. *Bifidobacterium asteroides* ESL017/ESL0822 and *Bifidobacterium* sp2. ESL0200/ESL0819; Fig. 3, Supplementary Fig. S10), whereas others, in particular *Lactobacillus apis* ESL0263, were only marginally affected. A GLMM confirmed that strains arriving second showed significantly reduced absolute abundance, relative to arriving first or as the only member of their species (*P* < .05 for second and *P* > .05 for first) for all strains except *Lactobacillus apis* ESL0263 (Supplementary Table S5). To quantify differences among strains, we calculated the priority effect strength in each community combination for a given strain as the log ratio of the median abundance of the strain when it was a firstcomer vs. latecomer (Fig. 1B, Supplementary Table S4). As expected, priority effect strength was higher than zero (i.e. log10 transformed value of 1) for all strains (Fig. 4), but varied across strains, even among those of the same species (Fig. 4). Moreover, some strains showed substantial variation across replicates, whereas others did not, suggesting that in some cases the community background, and hence the interactions with allospecific strains, influenced priority effect strength.

### Conspecifics account for only part of the observed priority effects

**Figure 5:**
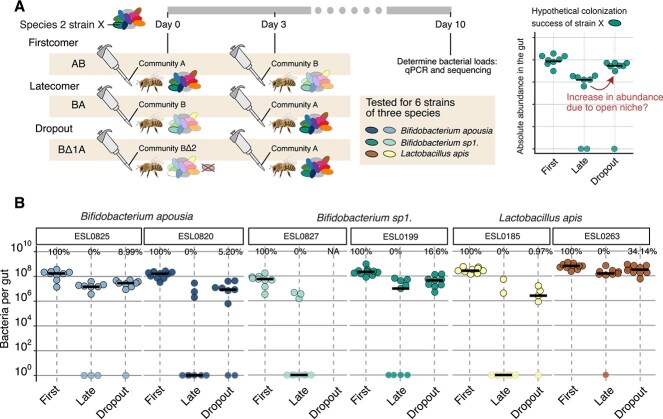
Colonization success of strains as latecomers in full and dropout treatments. (A) Scheme of the experimental design (left panel) and theoretical example of the analysis (right panel). (B) Absolute abundance of six tested strains of three species when arriving first (“First”) or late (“Late”) in the full community versus when arriving late in the dropout treatment (“Dropout”) in which the conspecific strain was dropped out from the firstcomer community. The percentages indicate the median colonization level of the focal strain relative to the median colonization level in the firstcomer community (100%) and the latecomer community (0%) using the formula: (abundance(Dropout) – abundance(Late)) / (abundance(First) – abundance(Late)). Only samples in which the strain was above the detection limit were considered for this calculation. The effect of the conspecific dropout on the abundance of strain ESL0827 of *Bifidobacterium sp1.* could not be determined due to low sequencing depth (Supplementary Fig. S3). Bee illustrations in this figure were created with BioRender.com.

We hypothesized that priority effects are primarily mediated by within-species (conspecific) competition, rather than between-species (allospecific) interactions, based on the assumption that conspecific strains exhibit greater niche overlap. To test this, we used the same experimental setup as before but modified the firstcomer community by omitting the strain of one focal species. At Day 3, we introduced the full latecomer community (Fig. 5A). This experiment was performed using three randomly selected species from the original set of 12 (*Bifidobacterium apousia*, *Bifidobacterium* sp1., and *Lactobacillus apis*), for which both strains were removed in reciprocal order (i.e. dropping out the firstcomer strain in the AB and BA treatment, respectively). To assess how the absence of the firstcomer strain affected colonization of the conspecific latecomer strain, we compared its absolute abundance in the dropout treatment to its abundance when introduced alongside the full community (either as firstcomer or latecomer). We expected that removing the firstcomer strain would increase colonization success of the latecomer conspecific strain, compared to the condition in which the full firstcomer community was present (Fig. 5A).

All latecomer strains, except for ESL0185 of *Lactobacillus apis*, showed improved colonization success when bees were colonized with the dropout firstcomer community compared to the full firstcomer community (Fig. 5B, “late” vs. “dropout”). However, none of the latecomer strains reached the same abundance levels as when they were introduced as part of the firstcomer community (Fig. 5B, “first”): they reached between 1% and 50% of the median abundance when introduced with the firstcomer community depending on the strain. Our sequencing analysis also revealed that latecomer strains of species other than the one that dropped out were affected. In particular, several *Bifidobacterium* species in treatments where the dropouts were *Bifidobacterium* strains (Fig. 5, Supplementary Table S6), were more successful in colonization in those two treatments than in the full community or other dropout treatments (Fig. 5, Supplementary Table S6). Based on these results, we conclude that priority effects are influenced not only by strain interactions within species (i.e. conspecific interactions) but also by interactions between species, particularly among closely related species.

## Discussion

Evidence from empirical studies is beginning to show that the order of arrival (i.e. priority effects) plays an important role in shaping community assembly in microbial ecosystems [15, 37–40] including gut microbiomes across a wide range of hosts, from mammals to fruit flies, honeybees, and *Daphnia* [18, 20–22, 41–43]. However, few studies have explored how priority effects influence the assembly of gut communities at the strain level within the context of defined multi-species microbial consortia. By leveraging full-length 16S rRNA gene amplicon sequencing to distinguish between conspecific strains, we demonstrate that priority effects are an important process shaping strain-level community assembly in the honeybee gut microbiota model.

We assessed the importance of priority effects by using a synthetic microbial community composed of several related species rather than individual strains [44]. This approach ensured that the observed effects were relevant in the presence of inter-species interactions, such as competition for space and nutrients, mimicking the natural environment in the gut (Brochet *et al.* [21]; Ghoul and Mitri [45]; Kešnerová *et al.* [34]). Further, our approach enabled us to simultaneously evaluate the impact of arrival order on several strains (two strains of eight species, i.e. a total of 16 strains) inoculated reciprocally as firstcomers and latecomers. To account for unexpected effects of interactions between allospecific strains in the same community, we tested several community combinations; specifically, the priority effect of each strain was assessed in the background of different allospecific strains.

Our findings demonstrate that priority effects can play a significant role in shaping the strain level composition of the bee gut microbiota. When bees were sequentially inoculated with the two communities, their gut microbiota consistently resembled that of bees exposed only to the first community. This indicates that firstcomer strains effectively dominated the community, hindering the establishment of latecomer strains. Such priority effects may explain-or at least contribute to-the natural variation in strain-level composition observed among individual worker bees within a colony [1]. Similar early-life effects have been shown to drive microbiota variation in laboratory mouse models, even under tightly controlled host and environmental conditions (Martínez *et al.* [17]). Like in other animals', the gut microbiota of adult bees is acquired after “birth” (i.e. pupal eclosion) through social interactions with nestmates. Therefore, the specific strains a newly emerged bee acquires likely depend on which nestmates it encounters during its early post-emergence period (firstcomer “seeding”). These initial interactions can thus result in persistent individual differences in microbiota composition and could even contribute to the host-specific nature of the bee gut microbiota [46]. This could be further tested in a natural setting by using tagged bacterial strains and tracking the interactions of newly emerged bees to compare the strain-level composition of their gut microbiota with that of the adults that they interacted with first as opposed to later in life.

Compared to other animals gut microbiomes, priority effects may be particularly strong in honeybees, as newly emerged adults spend the first few days inside the hive and only sporadically embark on defecation flights [47]. As a result, food residence time in the gut may be relatively long, potentially leading to low strain-level turnover, and ecological opportunities for exposure to the microbiota of other honeybee species are low. However, as adult worker bees age, they transition from in-hive nurses to outside foragers, undergoing substantial physiological and behavioral changes. During this shift they reduce pollen intake and increasingly rely on nectar and honey as their primary diet. Recent findings show that foragers harbor a distinct strain-level community compared to nurses [48]. Therefore, even though early arriving strains might dominate the community in nurse bees, other processes and mechanisms than priority effects are likely at play, such as selection in the host gut environment or dietary differences, and may affect the community composition, e.g. when bees transition to foragers.

Strains of the same species in the bee gut can differ substantially in their accessory gene content, leading to variation in functional potential [1, 28, 32, 49]. As a result, they may influence the host in distinct ways, e.g., through differences in carbohydrate degradation or the production of metabolites with neuroactive properties [50, 51]. Consequently, the order in which strains colonize the gut early in life, and the resulting differences in community composition at the strain-level, could have effects on how the microbiota impact the host. Such host effects of arrival order have been shown to occur in legume-rhizobium mutualism [38] and may likely also play an important role in more complex microbial communities, such as those in the animal gut. Our results indicate that the timing of colonization is an important consideration in the development of probiotic strategies based on bee gut bacteria aimed at improving bee health [52].

Although we found that arrival order influenced colonization outcomes for all tested community members, the strength of priority effects varied across strains. For instance, all strains of *B. asteroides*, *Bifidobacterium* sp2., *Lactobacillus helsingborgensis*, and *Lactobacillus kullabergensis* were almost entirely undetectable when introduced as part of the latecomer community. In contrast, strains from other species were still able to establish as latecomers, albeit at much lower abundances than when introduced first. The strain ESL0263 of *Lactobacillus apis* stood out for its exceptional ability to successfully colonize even as a latecomer. The underlying reasons for these differences among strains remain to be elucidated, but may involve variation in niche overlap or the ability to interfere with established strains. Further investigation into the mechanisms behind these differences could reveal valuable traits for probiotic development or microbial engineering. These approaches are also of growing interest in promoting the health of managed honeybee populations [53].

A previous study demonstrated that phylogenetic relatedness predicts the extent of priority effects among microbes [37], which means that priority effects were stronger between closer relatives, likely because they harbor greater niche overlap. Here, we tested this using dropout communities where one firstcomer strain was dropped at a time. Our expectation was that the latecomer strain of the same species would increase in colonization if the priority effect was mediated through intra-specific competition. Although this was the case for at least four of the six tested strains, we noted that the effect was relatively small (8%–34%). In other words, the latecomer strain did not reach the same abundance as when arriving in the firstcomer community. Moreover, several strains of other species in the latecomer communities increased in abundance when a strain was dropped out. These results suggest that priority effects are also mediated through interspecific interactions, in particular between species belonging to the same genus as the one dropped out. Hence, we hypothesize that not only strains of the same species but also those of different but closely related species have considerable niche overlap, and hence contribute to priority effects via niche pre-emption [54]. Niche overlap can arise from competition for both space and nutrients. In the bee gut, both processes may be at play. Previous work has shown that a diverse diet promotes the coexistence of divergent strains of major bee gut symbionts, suggesting resource-based niche partitioning [31, 55]. At the same time, bacterial taxa are known to colonize spatially restricted regions of the bee gut, indicating that physical space may also represent a limiting niche dimension [30, 56]. Measuring the metabolic capabilities of the strains could be used to determine the niche overlap in terms of nutrient utilization and subsequently predict community assembly and invasion. This was recently demonstrated in a study which showed that pathogens of the genera *Klebsiella* and *Salmonella* were less successful in invading communities containing species closely related to the pathogen *in vitro* and *in vivo* in the mouse gut [57]. Competition for space could be visualized using fluorescent tags in combination with microscopy as recently done for the priority effect observed for *S. alvi* in the bee gut [22] or for *Lactiplantibacillus plantarum* in the fruit fly gut [41].

Our findings underscore the importance of arrival order in shaping gut microbial communities, particularly at the strain level and for the assembly of the gut microbiota of honeybees. Although natural systems may buffer or modulate these effects through increased diversity and environmental complexity, the foundational role of early colonizers in determining community trajectories remains evident. These insights contribute to a broader understanding of how host-associated microbial communities are structured and maintained, with implications for both microbiome engineering in the context of bee health and ecological theory.

## Supplementary Material

260204_SupplementaryMaterial_R2_clean_wrag056

SupplementaryTables_R1_wrag056

## Data Availability

Raw reads are deposited in the NCBI SRA database under the Project ID PRJNA1289778 (https://www.ncbi.nlm.nih.gov/bioproject/PRJNA1289778/). Several intermediate files, including 16S rRNA gene sequence database and RDS objects for analysis are included in the GitHub repository: https://github.com/Aiswarya-prasad/20240399_aprasad_PriorityEffects.

## Code availability

All the code and intermediate files, including RDS objects, are found in the GitHub repository: https://github.com/Aiswarya-prasad/20240399_aprasad_PriorityEffects.

## References

[1] Ellegaard KM, Engel P. Genomic diversity landscape of the honey bee gut microbiota. *Nat Commun* 2019;10:446. 10.1038/s41467-019-08303-0 30683856 PMC6347622

[2] Garud NR, Good BH, Hallatschek O et al. Evolutionary dynamics of bacteria in the gut microbiome within and across hosts. *PLoS Biol* 2019;17:e3000102. 10.1371/journal.pbio.3000102 30673701 PMC6361464

[3] Truong DT, Tett A, Pasolli E et al. Microbial strain-level population structure and genetic diversity from metagenomes. *Genome Res* 2017;27:626–38. 10.1101/gr.216242.116 28167665 PMC5378180

[4] Wolff R, Shoemaker W, Garud N. Ecological stability emerges at the level of strains in the human gut microbiome. *MBio* 2023;14:e0250222–2. 10.1128/mbio.02502-22 36809109 PMC10127601

[5] Yan Y, Nguyen LH, Franzosa EA et al. Strain-level epidemiology of microbial communities and the human microbiome. *Genome Med* 2020;12:71. 10.1186/s13073-020-00765-y 32791981 PMC7427293

[6] Mallott EK, Amato KR. Host specificity of the gut microbiome. *Nat Rev Microbiol* 2021;19:639–53. 10.1038/s41579-021-00562-3 34045709

[7] Miller BM, Bäumler AJ. The habitat filters of microbiota-nourishing immunity. *Annu Rev Immunol* 2021;39:1–18. 10.1146/annurev-immunol-101819-024945 33902314

[8] Sprockett DD, Price JD, Juritsch AF et al. Home-site advantage for host species–specific gut microbiota. *Sci Adv* 2023;9:eadf5499. 10.1126/sciadv.adf5499 37184968 PMC10184861

[9] Mazel F, Guisan A, Parfrey LW. Transmission mode and dispersal traits correlate with host specificity in mammalian gut microbes. *Mol Ecol* 2023;33:e16862. 10.1111/mec.16862 36786039

[10] Moeller AH, Suzuki TA, Lin D et al. Dispersal limitation promotes the diversification of the mammalian gut microbiota. *Proc Natl Acad Sci* 2017;114:13768–73. 10.1073/pnas.1700122114 29229828 PMC5748161

[11] Nemergut DR, Schmidt SK, Fukami T et al. Patterns and processes of microbial community assembly. *Microbiol Mol Biol Rev* 2013;77:342–56. 10.1128/MMBR.00051-12 24006468 PMC3811611

[12] Zhou J, Ning D. Stochastic community assembly: does it matter in microbial ecology? *Microbiol Mol Biol Rev* 2017;81:10.1128. 10.1128/mmbr.00002-17 PMC5706748 29021219

[13] Debray R, Herbert RA, Jaffe AL et al. Priority effects in microbiome assembly. *Nat Rev Microbiol* 2022;20:109–21. 10.1038/s41579-021-00604-w 34453137

[14] Gleason HA . Further views on the succession-concept. *Ecology* 1927;8:299–326. 10.2307/1929332

[15] Carlström CI, Field CM, Bortfeld-Miller M et al. Synthetic microbiota reveal priority effects and keystone strains in the Arabidopsis phyllosphere. *Nat Ecol Evol* 2019;3:1445–54. 10.1038/s41559-019-0994-z 31558832 PMC6774761

[16] Jones KR, Belden LK, Hughey MC. Priority effects alter microbiome composition and increase abundance of probiotic taxa in treefrog tadpoles. *Appl Environ Microbiol* 2024;90:e0061924–4. 10.1128/aem.00619-24 38757977 PMC11218634

[17] Martínez I, Maldonado-Gomez MX, Gomes-Neto JC et al. Experimental evaluation of the importance of colonization history in early-life gut microbiota assembly. *elife* 2018;7:e36521. 10.7554/eLife.36521 30226190 PMC6143339

[18] Ojima MN, Jiang L, Arzamasov AA et al. Priority effects shape the structure of infant-type Bifidobacterium communities on human milk oligosaccharides. *ISME J* 2022;16:2265–79. 10.1038/s41396-022-01270-3 35768643 PMC9381805

[19] Sprockett D, Fukami T, Relman DA. Role of priority effects in the early-life assembly of the gut microbiota. *Nat Rev Gastroenterol Hepatol* 2018;15:197–205. 10.1038/nrgastro.2017.173 29362469 PMC6813786

[20] Chen JZ, Junker A, Zheng I et al. A strong priority effect in the assembly of a specialized insect-microbe symbiosis. *Appl Environ Microbiol* 2024;90:e0081824–4. 10.1128/aem.00818-24 39291984 PMC11497811

[21] Segura Munoz RR, Mantz S, Martínez I et al. Experimental evaluation of ecological principles to understand and modulate the outcome of bacterial strain competition in gut microbiomes. *ISME J* 2022;16:1594–604. 10.1038/s41396-022-01208-9 35210551 PMC9122919

[22] Jones KR, Song Y, Rinaldi SS et al. Effects of priority on strain-level composition of the honey bee gut community. *Appl Environ Microbiol* 2025;91:e0082825–5. 10.1128/aem.00828-25 40742109 PMC12366320

[23] Adler PB, HilleRisLambers J, Levine JM. A niche for neutrality. *Ecol Lett* 2007;10:95–104. 10.1111/j.1461-0248.2006.00996.x 17257097

[24] Chesson P . Mechanisms of maintenance of species diversity. *Annu Rev Ecol Syst* 2000;31:343–66. 10.1146/annurev.ecolsys.31.1.343

[25] Fukami T, Mordecai EA, Ostling A. A framework for priority effects. *J Veg Sci* 2016;27:655–7. 10.1111/jvs.12434

[26] Mayfield MM, Levine JM. Opposing effects of competitive exclusion on the phylogenetic structure of communities: phylogeny and coexistence. *Ecol Lett* 2010;13:1085–93. 10.1111/j.1461-0248.2010.01509.x 20576030

[27] Orr JA, Armitage DW, Letten AD. Coexistence theory for microbial ecology, and vice versa. *Environ Microbiol* 2025;27:e70072. 10.1111/1462-2920.70072 40033656 PMC11876725

[28] Engel P, Martinson VG, Moran NA. Functional diversity within the simple gut microbiota of the honey bee. *Proc Natl Acad Sci USA* 2012;109:11002–7. 10.1073/pnas.1202970109 22711827 PMC3390884

[29] Kwong WK, Moran NA. Gut microbial communities of social bees. *Nat Rev Microbiol* 2016;14:374–84. 10.1038/nrmicro.2016.43 27140688 PMC5648345

[30] Engel P, Bartlett KD, Moran NA. The bacterium Frischella perrara causes scab formation in the gut of its honeybee host. *MBio* 2015;6:e00193–15. 10.1128/mBio.00193-15 25991680 PMC4442143

[31] Brochet S, Quinn A, Mars RAT et al. Niche partitioning facilitates coexistence of closely related honey bee gut bacteria. *elife* 2021;10:e68583. 10.7554/eLife.68583 34279218 PMC8456714

[32] Ellegaard KM, Brochet S, Bonilla-Rosso G et al. Genomic changes underlying host specialization in the bee gut symbiont *lactobacillus* Firm5. *Mol Ecol* 2019;28:2224–37. 10.1111/mec.15075 30864192

[33] Seemann T . Barrnap 0.9: Rapid Ribosomal RNA Prediction, Vol. 2013, 2013. https://github.com/tseemann/barrnap

[34] Kešnerová L, Mars RAT, Ellegaard KM et al. Disentangling metabolic functions of bacteria in the honey bee gut. *PLoS Biol* 2017;15:e2003467. 10.1371/journal.pbio.2003467 29232373 PMC5726620

[35] Callahan BJ, McMurdie PJ, Rosen MJ et al. DADA2: high-resolution sample inference from Illumina amplicon data. *Nat Methods* 2016;13:581–3. 10.1038/nmeth.3869 27214047 PMC4927377

[36] Callahan BJ, Wong J, Heiner C et al. High-throughput amplicon sequencing of the full-length 16S rRNA gene with single-nucleotide resolution. *Nucleic Acids Res* 2019;47:e103. 10.1093/nar/gkz569 31269198 PMC6765137

[37] Peay KG, Belisle M, Fukami T. Phylogenetic relatedness predicts priority effects in nectar yeast communities. *Proc R Soc B Biol Sci* 2011;279:749–58. 10.1098/rspb.2011.1230 PMC3248732 21775330

[38] Boyle JA, Simonsen AK, Frederickson ME et al. Priority effects alter interaction outcomes in a legume–rhizobium mutualism. *Proc R Soc B Biol Sci* 2021;288:20202753. 10.1098/rspb.2020.2753 PMC7944086 33715440

[39] Debray R, Conover A, Zhang X et al. Within-host adaptation alters priority effects within the tomato phyllosphere microbiome. *Nat Ecol Evol* 2023;7:725–31. 10.1038/s41559-023-02040-w 37055621

[40] Garrido-Sanz D, Keel C. Seed-borne bacteria drive wheat rhizosphere microbiome assembly via niche partitioning and facilitation. Nat Microbiol 2025;10:1130–44. 10.1038/s41564-025-01973-1 PMC12055584 40140705

[41] Dodge R, Jones EW, Zhu H et al. A symbiotic physical niche in Drosophila melanogaster regulates stable association of a multi-species gut microbiota. *Nat Commun* 2023;14:1557. 10.1038/s41467-023-36942-x 36944617 PMC10030875

[42] Gurung A, Mukherjee S, Declercq M et al. Strain-dependent and host genotype–dependent priority effects in gut microbiome assembly affect host fitness in. *Limnol Oceanogr* 2024;69:1782–96. 10.1002/lno.12614

[43] Laursen MF, Roager HM. Human milk oligosaccharides modify the strength of priority effects in the Bifidobacterium community assembly during infancy. *ISME J* 2023;17:2452–7. 10.1038/s41396-023-01525-7 37816852 PMC10689826

[44] Großkopf T, Soyer OS. Synthetic microbial communities. *Curr Opin Microbiol* 2014;18:72–7. 10.1016/j.mib.2014.02.002 24632350 PMC4005913

[45] Ghoul M, Mitri S . The ecology and evolution of microbial competition. *Trends in Microbiol* 2016;24:833–45. 10.1016/j.tim.2016.06.011 27546832

[46] Mazel F, Prasad A, Engel P. Host specificity of gut microbiota associated with social bees: patterns and processes. *Microbiol Mol Biol Rev* 2025;89:e0008023–3. 10.1128/mmbr.00080-23 40111037 PMC12188721

[47] LL Langstroth Lorenzo L. Langstroth on the Hive and the Honey-Bee, a Bee-keeper’s Manual. Northampton, 1853, Hopkins, Bridgman, 10.5962/bhl.title.38048.

[48] Baud GLC, Prasad A, Ellegaard KM et al. Turnover of strain-level diversity modulates functional traits in the honeybee gut microbiome between nurses and foragers. *Genome Biol* 2023;24:283. 10.1186/s13059-023-03131-4 38066630 PMC10704631

[49] Van Rossum T, Ferretti P, Maistrenko OM et al. Diversity within species: interpreting strains in microbiomes. *Nat Rev Microbiol* 2020;18:491–506. 10.1038/s41579-020-0368-1 32499497 PMC7610499

[50] Cabirol A, Moriano-Gutierrez S, Engel P. Neuroactive metabolites modulated by the gut microbiota in honey bees. *Mol Microbiol* 2024;122:284–93. 10.1111/mmi.15167 37718573

[51] Zheng H, Perreau J, Powell JE et al. Division of labor in honey bee gut microbiota for plant polysaccharide digestion. *Proc Natl Acad Sci USA* 2019;116:25909–16. 10.1073/pnas.1916224116 31776248 PMC6926048

[52] Damico ME, Beasley B, Greenstein D et al. Testing the effectiveness of a commercially sold probiotic on restoring the gut microbiota of honey bees: a Field study. *Probiotics & Antimicro Prot* 2025;17:991–1000. 10.1007/s12602-023-10203-1 PMC12055933 38112994

[53] Motta EVS, Powell JE, Leonard SP et al. Prospects for probiotics in social bees. *Philosophical Transactions of the Royal Society B: Biological Sciences* 2022;377:20210156. 10.1098/rstb.2021.0156 PMC9058534 35491599

[54] Levy R, Borenstein E. Metabolic modeling of species interaction in the human microbiome elucidates community-level assembly rules. *Proc Natl Acad Sci* 2013;110:12804–9. 10.1073/pnas.1300926110 23858463 PMC3732988

[55] Yang C, Han B, Tang J et al. Life history strategies complement niche partitioning to support the coexistence of closely related Gilliamella species in the bee gut. *ISME J* 2025;19:wraf016. 10.1093/ismejo/wraf016 39893622 PMC11822680

[56] Li Y, Leonard SP, Powell JE et al. Species divergence in gut-restricted bacteria of social bees. *Proc Natl Acad Sci USA* 2022;119:e2115013119. 10.1073/pnas.2115013119 35467987 PMC9170019

[57] Spragge F, Bakkeren E, Jahn MT et al. Microbiome diversity protects against pathogens by nutrient blocking. *Science* 2023;382:eadj3502. 10.1126/science.adj3502 38096285 PMC7616675

